# Telemedicine-Based Approach for Obstructive Sleep Apnea Management: Building Evidence

**DOI:** 10.2196/ijmr.3060

**Published:** 2014-02-19

**Authors:** Valentina Isetta, Carmen León, Marta Torres, Cristina Embid, Josep Roca, Daniel Navajas, Ramon Farré, Josep M Montserrat

**Affiliations:** ^1^Unit of Biophysics and BioengineeringFaculty of MedicineUniversity of BarcelonaBarcelonaSpain; ^2^CIBER de Enfermedades Respiratorias (CIBERES)BunyolaSpain; ^3^Sleep LaboratoryPneumology DepartmentHospital Clínic of BarcelonaBarcelonaSpain; ^4^Pneumology DepartmentHospital Clínic of BarcelonaBarcelonaSpain; ^5^Institut d’Investigacions Biomediques August Pi Sunyer (IDIBAPS)BarcelonaSpain; ^6^Institut de Bioenginyeria de Catalunya (IBEC)BarcelonaSpain

**Keywords:** telemedicine, sleep apnea, CPAP therapy, teleconsultation

## Abstract

**Background:**

Telemedicine seems to offer reliable solutions to health care challenges, but significant contradictory results were recently found. Therefore, it is crucial to carefully select outcomes and target patients who may take advantage of this technology. Continuous positive airway pressure (CPAP) therapy compliance is essential to treat patients with obstructive sleep apnea (OSA). We believe that OSA patients could benefit greatly from a telemedicine approach for CPAP therapy management.

**Objective:**

The objective of our study was to evaluate the application of a telemedicine-based approach in the CPAP therapy management, focusing on patients’ CPAP follow-up and training.

**Methods:**

We performed two studies. First, (study 1) we enrolled 50 consecutive OSA patients who came to our sleep center for the CPAP follow-up visit. Patients performed a teleconsultation with a physician, and once finalized, they were asked to answer anonymously to a questionnaire regarding their opinion about the teleconsultation. In a second randomized controlled trial (RCT) (study 2), we included 40 OSA patients scheduled for CPAP training. There were 20 that received the usual face-to-face training and 20 that received the training via videoconference. After the session, they were blindly evaluated on what they learned about OSA and mask placement.

**Results:**

More than 95% (49/50) of the interviewed patients were satisfied with the teleconsultation, and 66% (33/50) of them answered that the teleconsultation could replace 50%-100% of their CPAP follow-up visits. Regarding the RCT, patients who received the CPAP training via videoconference demonstrated the same knowledge about OSA and CPAP therapy as the face-to-face group (mean 93.6% of correct answers vs mean 92.1%; *P*=.935). Performance on practical skills (mask and headgear placement, leaks avoidance) was also similar between the two groups.

**Conclusions:**

OSA patients gave a positive feedback about the use of teleconsultation for CPAP follow-up, and the CPAP training based on a telemedicine approach proved to be as effective as face-to-face training. These results support the use of this telemedicine-based approach as a valuable strategy for patients’ CPAP training and clinical follow-up.

## Introduction

### The New Challenge of Telemedicine

Born as a method to deliver health care at a distance, telemedicine seems to offer credible solutions, tested in real medical settings, to the main challenges facing our society, such as population aging, chronic patients’ management, and health costs reduction. However, recently published results questioned the validity of this technology as a health care delivery method for all populations [1]. Consequently, it appears crucial to carefully select proper outcomes and most receptive target patients’ groups.

### Obstructive Sleep Apnea in the Population

Obstructive sleep apnea (OSA) is a very prevalent disorder that is estimated to affect 2%-4% of adult men and 1%-2% of adult women in Western countries [2,3]. OSA entails repetitive obstructions of the upper airway resulting in brain arousal, intermittent hypoxia, large negative intrathoracic pressures, and increased sympathetic activation. All these phenomena induce intermediate systemic changes such as inflammation, oxidative stress, or metabolic changes that result in different clinical symptoms, including fatigue and daytime somnolence [4]. Recently, the awareness of OSA in the media and in medical and patients’ circles has exponentially increased, and therefore the number of patients for evaluation. At present, OSA is considered as a major factor responsible for different cardiovascular, neurologic, and metabolic diseases. In addition, OSA prevalence is likely to increase due to its strong association to obesity, which is considered epidemic currently, and for its higher incidence in the aging population, another rising part of the population. Finally, there is a growing evidence of OSA as a risk factor for traffic accidents [5].

This increase in demand, however, has not been accompanied by any improvements to deal with this problem. The European and Spanish public health resources assigned to sleep related breathing disorders have proved to be relatively inadequate and unlikely to handle the increase in OSA cases [6], therefore alternative and cost-effective management approaches are needed.

### Continuous Positive Airway Pressure Treatment

The treatment of choice for OSA is continuous positive airway pressure (CPAP) applied usually through a mask to the patient’s nose during sleep. This pressure in the mask is transmitted to the pharyngeal area, thereby avoiding upper airway obstruction. CPAP therapy compliance is essential to guarantee its effectiveness to treat OSA patients [7]. Despite the documented clinical efficacy of CPAP, it is estimated that 30% up to 80% of patients underuse or even suspend CPAP treatment [8], mainly due to its discomforting side effects and lack of improvement perception. Main CPAP therapy side effects are pressure intolerance, claustrophobic reaction to the mask, mask displacement, and machine noise [9], which may even disrupt sleep or provoke hypertension at night [10]. Many of these problems could be solved by a closer follow-up, but the overloaded sleep centers have troubles to provide such support.

Therefore, innovative interventions are needed to enhance the CPAP compliance, especially in the first few months of treatment when the long-term compliance level of a patient is usually defined [11]. In addition, it is worth noting the great socioeconomic impact of CPAP treatment, whose prevalence is estimated at 0.6% up to 2% of the entire population [12,13], being two-thirds of all respiratory therapies provided at home [14]. Consequently, we believe that a telemedicine-based approach for CPAP therapy management could be of great interest and benefit for OSA patients.

### A Telemedicine-Based Approach for Continuous Positive Airway Pressure Treatment

Our aim was to evaluate the application of a telemedicine-based approach in CPAP therapy management, particularly focusing on the application of teleconsultation, that is, a medical visit via videoconference, to patients’ CPAP follow-up and training. In a previous study, we received positive feedback from a group of physicians who declared that teleconsultation could avoid 45% of face-to-face follow-up visits [15]. The present work was focused on OSA patients, whose acceptance level and opinion for this specific application are still unclear. First, after performing a CPAP follow-up visit via videoconference, we asked a group of OSA patients to give us feedback about it. In a second randomized controlled trial (RCT), we performed a blinded comparison between the knowledge and skills of a group of OSA patients who received the standard face-to-face CPAP training and another group who received it via videoconference. The Hospital Clínic of Barcelona Ethics Committee authorized both studies.

## Methods

### Study 1: Continuous Positive Airway Pressure Follow-Up

We recruited 50 consecutive OSA patients who came to the Hospital Clínic sleep center for a routine CPAP therapy follow-up visit. After finalizing the visit, the nurse asked the patient to participate in the study. The exclusion criteria were illiteracy, deafness, and refusal to participate. Upon signature of the informed consent, the patient was accompanied to the next room, which was equipped with an Internet-connected personal computer (PC) with a web-cam and the free videoconference software Skype installed. The patient was connected with a physician who was waiting in his/her office. After checking the audio-video quality of the conference call, the patient was left alone for the interview with the physician. The brief consultation was composed of structured questions about the CPAP therapy and possible problems and discomforts. Once the teleconsultation finalized, the patient, still alone in the room, answered a multiple choice questionnaire regarding his/her opinion about the teleconsultation and then delivered it in a sealed envelope to keep it anonymous.

### Study 2: Continuous Positive Airway Pressure Training

We enrolled 40 consecutive recently diagnosed OSA patients who came to our sleep center for CPAP training before starting the therapy. In this second study, the exclusion criteria were the same as the first one–illiteracy, deafness, and refusal to participate. After signing the informed consent, they were randomized in two groups–20 patients received the usual face-to-face CPAP training, while the other 20 received it via videoconference. The training session was the same as it is usually performed in our hospital before starting the CPAP therapy, and the same specialized nurse carried it out for both groups. It consisted of a theoretical part, where the patient received education about the nature, complications, and treatment of OSA with CPAP, and a practical part, in which the nurse gave full training on CPAP machine functioning, mask and headgear placement, and strategies to avoid mask leaks. Then, the patient was left for a few minutes with the mask placed and a fixed air pressure to familiarize him or herself with the therapy.

Once the session was finalized, patients were blindly evaluated by an expert on what they had learned. First, they were asked to answer a multiple choice test to assess what they had learned during the theoretical part of the session about OSA and CPAP therapy (see Multimedia Appendix 1). Then, they were asked to perform three simple tasks: (1) putting the mask on themselves, (2) placing the headgear, and (3) achieving an absence of mask leaks. Their skills were blindly evaluated on the following discrete scale–0 (bad), 1 (average), and 2 (good).

### Statistical Analysis

The normal distributed continuous variables are shown as mean (SD). Discrete variables are presented as absolute and relative frequencies (percentages). The comparison of discrete variables was done through the χ2 test or the Fisher exact test. Comparisons of the groups for continuous variables were performed with the unpaired *t* test for independent samples or the Mann-Whitney Rank Sum test (when continuous variable was not normally distributed).

## Results

### Study 1: Continuous Positive Airway Pressure Follow-Up

Of the 50 consecutive patients approached, all of them were included in the study. Table 1 describes some demographic characteristics of the patients included. The frequency distribution of the answers to the satisfaction survey is summarized in Table 2. The majority of the patients answered positively to all questions. More than 95% (49/50) of the patients were satisfied with the teleconsultation, and 66% (33/50) of them answered that teleconsultation could replace between 50% and 100% of the CPAP therapy follow-up visits (questions A and B of Table 2). In addition, 80% (40/50) of the interviewed patients would recommend teleconsultation to others (question C of Table 2). It is also noteworthy that the majority of patients did not find any problem regarding the audio-video quality of the videoconference, comfort, and safety during the teleconsultation (questions D, E, and F of Table 2).

**Figure 1:**
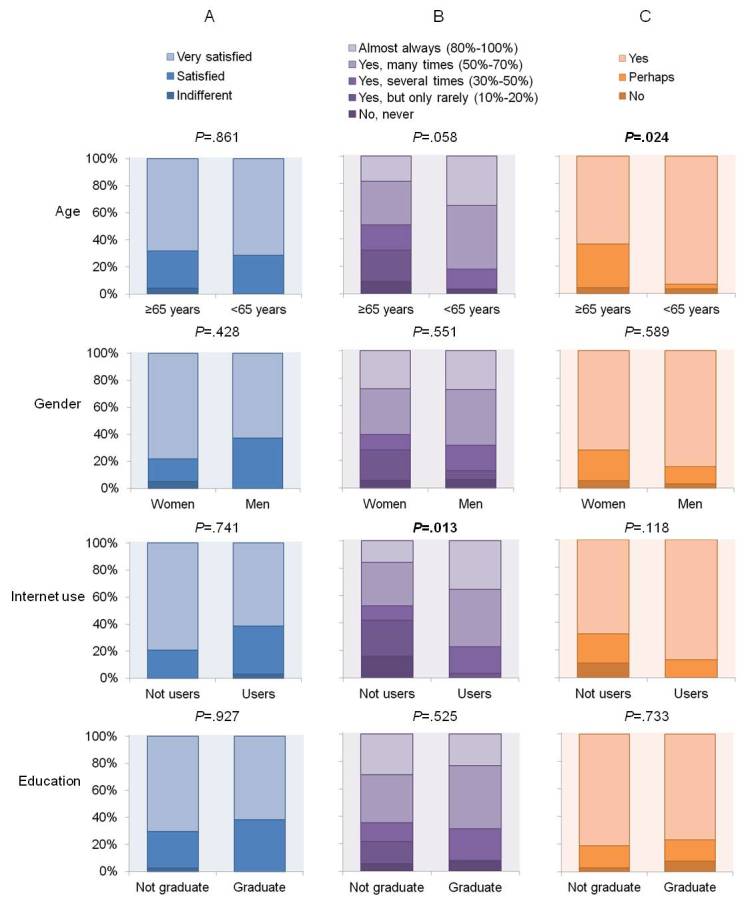
Frequency distribution of the answers to question A (satisfaction with the teleconsultation), B (percentage of face-to-face that could be replaced by teleconsultation), and C (inclination to recommend teleconsultation to others) of the opinion questionnaire stratified by age, gender, Internet use, and education (graduate studies or not) of the patients included in the first study. Significant differences between distributions (*P*<.05) are shown in italics.

Besides, we further analyzed the answers to the first three questions (A–satisfaction with the teleconsultation, B–percentage of face-to-face that teleconsultation could replace, and C–inclination to recommend teleconsultation to others) to assess the potential impact of some relevant population characteristics to the results. Accordingly, we stratified the interviewed population by gender, age, education, and Internet use, and we assessed the frequency distribution of the answers (Figure 1 shows the frequency distribution of Table 1, questions A, B, and C). Younger patients (<65 years) showed to be more inclined to recommend teleconsultation to others and Internet-users would replace a higher percentage of face-to-face follow-up visits with teleconsultation. Gender and education (graduated or not) seemed to have no impact to the patients’ opinion.

**Table 1:** Demographic characteristics of patients included in study 1 (n=50).

Demographic variable	Value
Male, (%)	64
Age–years, mean (SD)	62.1 (13.5)
BMI–mean (SD)	32.4 (6.4)
Graduate studies, (%)	26
Internet users, (%)	44

**Table 2:** Frequency distribution of the patients’ answers to the opinion questionnaire about the teleconsultation (study 1; n=50).

Question	Multiple choice answers	Results^a^ n (%)
1. Which is your level of satisfaction with the teleconsultation?	Very dissatisfied	0 (0)
	Dissatisfied	0 (0)
	Indifferent	1 (2)
	Satisfied	15 (30)
	*Very satisfied*	*34 (68)*
2. Do you believe that such teleconsultations could replace the follow-up visits to monitor your condition?	No, never	3 (6)
	Yes, but only rarely (10%-20%)	6 (12)
	Yes, several times (30%-50%)	8 (16)
	*Yes, many times (50%-70%)*	*19 (38)*
	Almost always (80%-100%)	14 (28)
3. Would you recommend this telemedicine system to others?	No	2 (4)
	Perhaps	8 (16)
	*Yes*	*40 (80)*
4. Could you hear and see well the doctor during the interview?	No, I could not	0 (0)
	Yes, with some problems	6 (12)
	*Yes, perfectly*	*43 (88)*
5. Did you feel comfortable during the interview?	No	1 (2)
	Quite	10 (20)
	*Very*	*39 (78)*
6. Did you feel safe about your privacy and confidentiality in the interview?	No	0 (0)
	Indifferent	4 (8)
	*Yes*	*46 (92)*

^a^For each question the highest frequencies are shown in italic.

### Study 2: Continuous Positive Airway Pressure Training

All of the 40 patients approached gave their consent to participate. The two randomized groups had similar demographic data (Table 3).

**Figure 2:**
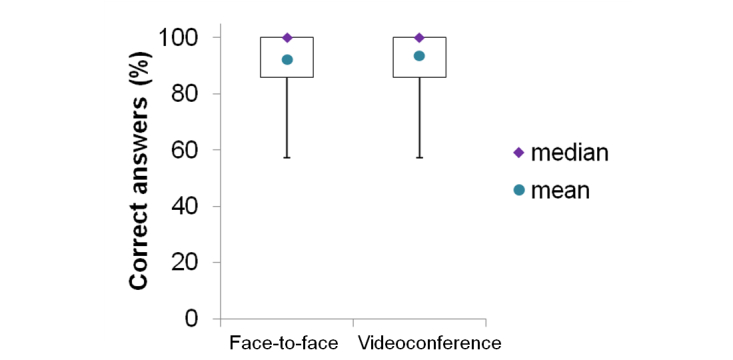
Results of the first part of patients’ evaluation regarding what they learned about OSA and CPAP therapy during the training session (study 2). The box-and-whickers plot shows the percentage of correct answers to the multiple choice test performed by the two randomized groups.

**Figure 3:**
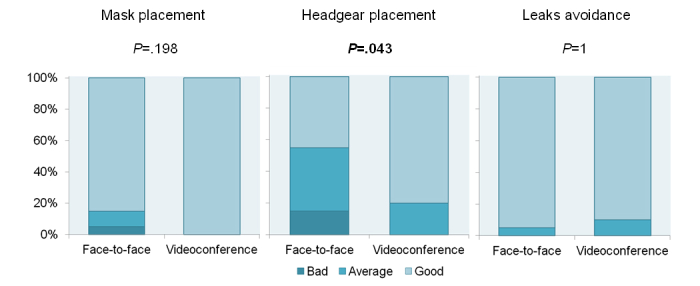
Results distribution of the practical evaluation of patients’ skills on placing the mask, the headgear, and avoiding leaks from the mask (study 2). A blinded expert scored patients’ performance on a ascendant scale: 0 (bad), 1 (average), and 2 (good).

Regarding the theoretical part of the evaluation, Figure 2 shows the percentage of correct answers to the multiple choice test of the two study groups. Patients who received the CPAP training via videoconference demonstrated the same knowledge about OSA and CPAP therapy as the face-to-face group (mean 93.6% of correct answers vs mean 92.1%; *P*=.935). Concerning the practical evaluation performed by the blinded expert, patients who received the CPAP training via videoconference showed similar performances to the ones who received the face-to-face training on mask placement (*P*=.198) and mask leaks avoidance (*P*=1.00). The videoconference group showed a slightly better performance in placing the headgear compared to the face-to-face group (*P*=.043). Figure 3 shows the results summarized graphically.

**Table 3:** Demographic characteristics of patients included in the RCT study (study 2; n=40).

	Face-to-face	Videoconference	*P*
n	20	20	
Male (%)	80	65	.480
Age years, mean (SD)	58.8 (12.8)	56.9 (8.9)	.587

## Discussion

### Principal Findings

In this paper, we assessed the potential usefulness of a telemedicine-based approach in the management of OSA patients under CPAP treatment. Specifically, we evaluated the application of videoconference to two essential phases of the OSA patients’ management–the CPAP therapy follow-up, and training. In the first study, we obtained a positive feedback from a group of 50 consecutive OSA patients who were interviewed after performing a CPAP follow-up visit via teleconsultation with a physician. Almost all of them were satisfied with this new approach and many would replace their usual face-to-face follow-up visit with teleconsultation, especially Internet users, and would recommend this approach to others, in particular, patients under 65 years old. In the second study, the two groups demonstrated similar results on what they learned during the CPAP training session in terms of both theoretical and practical skills, confirming the teleconsultation as effective as the classic face-to-face approach to deliver education and training to this kind of patient. To our knowledge, this is the first study in which a telemedicine-based approach for CPAP training is implemented and objectively evaluated in a RCT with OSA patients.

### Comparison With Previous Studies

In the last decades, it has been shown that simple telemedicine interventions, such as weekly phone calls to clarify doubts and encourage CPAP use, can markedly improve compliance [16]. An RCT showed that the use of a telephone-linked communication system providing feedback and counseling to OSA patients at home improved CPAP adherence, patients’ functional status, and reduced symptoms of depression [17]. Furthermore, another previous study employed an Internet-based informational support service for problems experienced with CPAP use [18]. Despite the organizational limitations and the poor differences between intervention and control group follow-up, the authors obtained good patients’ acceptance on this monitoring approach.

Considering a more technical approach, an RCT study assessed the impact on CPAP compliance and OSA outcomes of a wireless telemonitoring of CPAP compliance and efficacy data, compared to usual clinical care [19]. In this study, the intervention group was equipped with a CPAP machine outfitted with a wireless transmitter, which allowed the remote data transmission of compliance and therapy efficacy information to a computer server. Physicians could access it and use this information for the management of the patients. In contrast with a previous pilot study [20], a significant difference in terms of CPAP compliance was found between the study groups after 3 months, though more technician time was spent on patients in the telemedicine arm, entailing an extra cost for this strategy. It is also remarkable that teleconsultations have been found to improve CPAP adherence in a small group of nonadherent patients versus a placebo-controlled group [21]. The cost of the interventions, including the telehealth monitor, home installation, and telephone charges, was lower than the same number of face-to-face visits. Nevertheless, larger studies are needed to generalize any conclusion.

More in general, videoconferencing has demonstrated to be a valuable means for delivering health care interventions for chronic patients. Results of several studies indicated that interventions for a variety of psychological and physical conditions delivered by videoconferencing produce similar outcomes to treatment delivered in-person and a high level of patients’ satisfaction [22]. In the present study, we chose to employ the free videoconference tool Skype for its fast availability, ease of use, and good performance. Even though the risks and benefits of the use of Skype for clinical purposes should be accurately assessed [23], relevant scientific circles have recently started to discuss the potential value of Skype in different clinical applications [24-27].

Recently, there is a growing awareness of the need of further analyses about health care strategies based on telemedicine, which is also justified by recently published results that questioned the validity of this technology as a health care delivery method. Takahashi et al [28] reported on the results of an RCT of telemonitoring in older adults at high risk for hospitalization. They found that in-home monitoring of biometrics (eg, blood pressure and weight) and symptoms failed to reduce hospital readmissions or the need for emergency department visits compared with usual care. A few months later another study ran into similar results [1]. The data of these reports are important to make thorough considerations about research on telemedicine and its future clinical applications. First, we need a better understanding of the factors that depend on patients, physicians, the health system, and telemedicine programs that predict success. This would allow us to target and customize these interventions to patients who are most likely to benefit from them. Moreover, we should carefully select the appropriate outcomes that telemedicine health care strategies seek to effect [29].

While patients’ perception of a telemedicine intervention is usually assessed, clinical staff’s opinion and acceptance level are often poorly analyzed. Although the few available data are encouraging, showing a good clinical staff’s acceptance level of telemedicine [30,31] and also of a specific application in OSA management [15], clinicians’ feedback should be further assessed and their involvement promoted as main actor to guarantee a successful telemedicine intervention.

OSA patients, for their characteristics and the chronic nature of their pathology, seem to be a population that could particularly benefit from a telemedicine-based approach for its management. As mentioned before, despite the previous encouraging results, the actual usefulness of telemedicine for OSA patients under CPAP treatment is still to be fully proved. With this work, we wanted to contribute with straightforward and practical solutions to provide reliable data to assess the actual impact of telemedicine on OSA management.

### Limitations

In the two studies described in this paper, patients performed the teleconsultation and the CPAP training via videoconference in a room of our outpatient clinic, which could be argued to be an unrealistic setting. For this reason, patients’ opinions about audio-video quality of the teleconsultation, comfort, and safety should be taken with caution, as this survey did not imply that the subjects would feel the same at home, with their own video/computer resources and uncontrolled environment. Nevertheless, the patients included in our studies were left alone in the room in front of an Internet-connected PC and did not receive any assistance during the videoconference, except being previously briefly informed about the use of the videoconference tool, which could be done by a family member or a technician at home. Thus, we believe that this did not influence the results of our study, whose aim was to gather objective and consistent data to evaluate the feasibility and patients’ opinion of this new OSA management approach.

### Future Directions

Despite the encouraging findings of this work, additional large multicenter randomized studies are needed to further clarify the role of telemedicine in OSA management. Besides assessing clinical important issues, such as the enhancement of CPAP adherence and patients’ quality of life, cost-effectiveness analysis should be performed. In fact, this new management strategy could potentially lead to cost savings by reducing face-to-face visits, training sessions, and extending access to them for big rural areas inhabitants, like in Australia or Canada.

Although positive results in terms of cost-effectiveness of telemedicine-based strategies have been found in several fields [32-34] and even in OSA diagnosis [35], these should be confirmed also for CPAP therapy management.

### Conclusions

The interviewed OSA patients gave a positive feedback about the use of teleconsultation for their clinical follow-up, as well as physicians did in our previous study [15]. For the first time in this study, to our knowledge, we implemented a telemedicine-based CPAP training and objectively evaluated its impact on patients. The CPAP training performed via videoconference proved to be as effective as the face-to-face training, confirming the teleconsultation as a useful tool to deliver education and training to OSA patients. Our results support the use of this telemedicine-based approach as a valuable strategy for the management of OSA patients under CPAP treatment, which is a particularly relevant issue from both a clinical and socioeconomic point of view.

## Multimedia Appendix 1

Multiple choice test to evaluate the patients’ knowledge after receiving the CPAP training session (either face-to-face or via videoconference).

## Abbreviations

CPAP: continuous positive airway pressure
OSA: obstructive sleep apnea
PC: personal computer
RCT: randomized controlled trial
SEPAR: Spanish Society of Pneumology
