# Supplementary figures and images for: Telemedicine-Based Approach for Obstructive Sleep Apnea Management: Building Evidence
Source: Interact J Med Res. 2014 Feb 19;3(1):e6. doi: 10.2196/ijmr.3060 (PMC3961625; doi:10.2196/ijmr.3060)

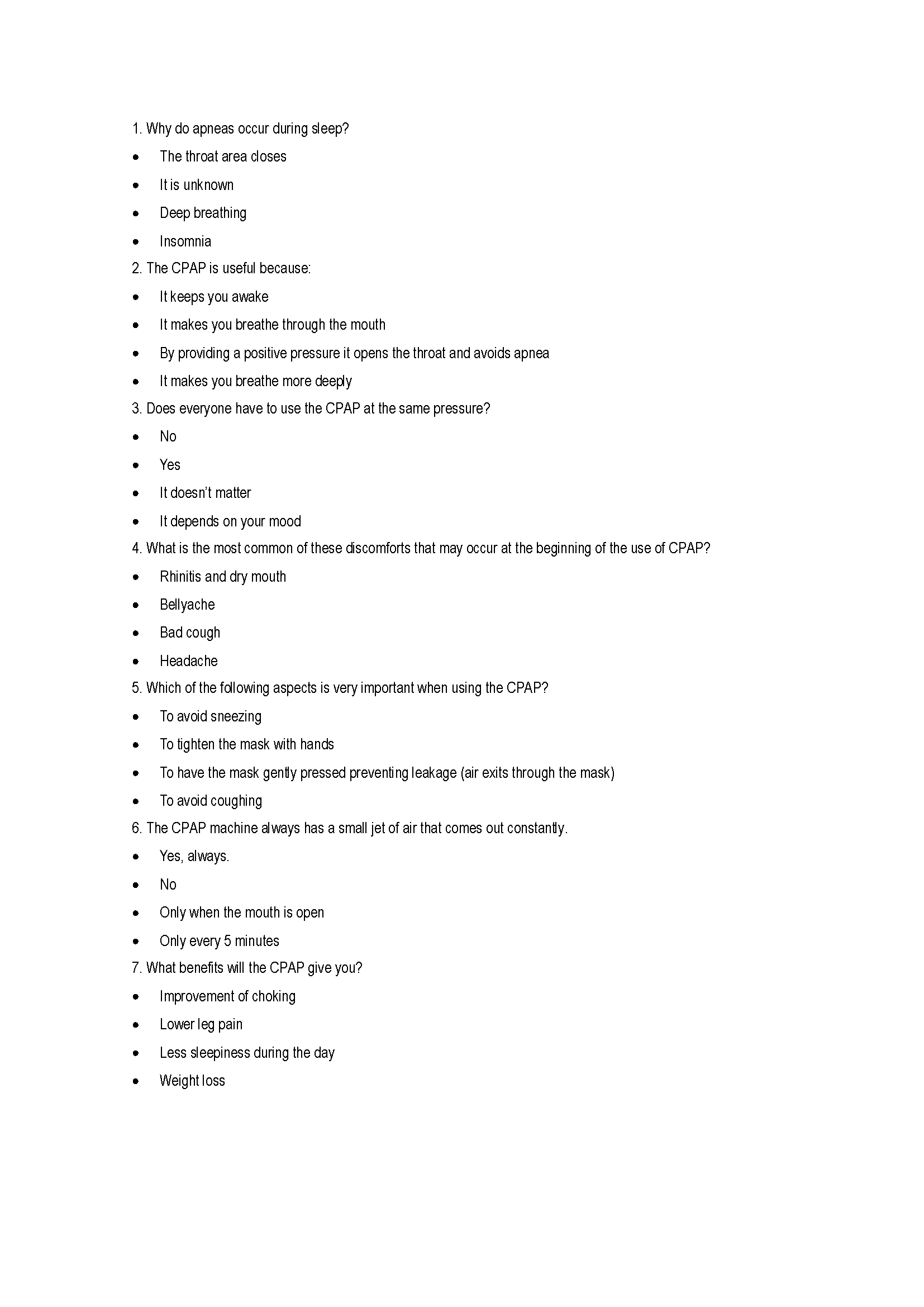

Supplement: Supplementary file 1 [file ijmr_v3i1e6_app1.png]
